# Predictive factors associated with axial length growth and myopia progression in orthokeratology

**DOI:** 10.1371/journal.pone.0218140

**Published:** 2019-06-12

**Authors:** Jaeryung Kim, Dong Hui Lim, Sun Hyup Han, Tae-Young Chung

**Affiliations:** Department of Ophthalmology, Samsung Medical Center, Sungkyunkwan University School of Medicine, Seoul, Republic of Korea; Keio University School of Medicine, JAPAN

## Abstract

**Purpose:**

To investigate the factors affecting axial length (AXL) growth and myopia progression in orthokeratology.

**Methods:**

This prospective, observational study enrolled 28 new orthokeratology lens wearers from a contact lens clinic between March 2016 and March 2017. Among them, 32 eyes of 17 wearers who completed one year of follow-up were finally analyzed. All participants underwent central (C) and peripheral (nasal 30° [N30] and temporal 30° [T30]) AXL measurements as well as central and peripheral refraction, ocular aberrations, and corneal topography at baseline and every posttreatment visit. A generalized estimating equation (GEE) was used to assess the associations between AXL change and all independent variables in both eyes.

**Results:**

The mean central AXL was 24.21 ± 0.60 mm and the mean baseline central spherical equivalent refractive error (SER) was −2.43 ± 0.97 diopters (D). Among all parameters that were significantly associated with AXL change in univariable GEE analyses, the baseline difference in AXL between C and N30 (β = −0.213, *p* < 0.001), baseline SER (β = −0.040, *p* < 0.033), posttreatment coma (β = −0.291, *p* < 0.031), third-order higher-order aberrations (HOAs) (β = −0.482, *p* < 0.001), and changes in second-order aberrations (β = 0.025, *p* = 0.027) at one year of follow-up were identified as significant factors in multivariable GEE analysis.

**Conclusions:**

The inhibition of AXL elongation and myopia progression in orthokeratology lens wear is significantly associated with the peripheral myopization and asymmetric optical changes mostly induced by third-order HOAs.

## Introduction

Myopia is one of the most common ocular diseases that can present during childhood. The prevalence of myopia has increased over the last few decades [1], especially in East Asians [2]. The progression of early-onset myopia is usually attributed to axial length (AXL) elongation that is not fully compensated for by a reduction in refractive power of the cornea and crystalline lens [3, 4]. Although myopia is usually adequately corrected by eyeglasses or contact lenses, these treatments cannot prevent AXL elongation. Because high myopia is closely associated with vision-threatening complications such as macular degeneration, retinal detachment, and glaucoma [5, 6], the hindering of myopia progression could potentially benefit children with myopia worldwide by ensuring a decreased risk of these complications.

Orthokeratology can be defined as a technique involving the programmed application of contact lenses to manipulate corneal curvature [7]. Ever since Wlodyga and Stoyan firstly designed a reverse geometry lens in 1989 [8], technological advances including the use of new lens materials with higher oxygen permeability and the incorporation of corneal topography to fit orthokeratology lenses and monitor changes in corneal shape have been made [7]. Following the first United States Food and Drug Administration approval of overnight orthokeratology lenses for all ages (Paragon CRT Contact Lenses; Paragon Vision Sciences, Mesa, AZ, USA), alleviating many of the safety concerns, orthokeratology has become increasingly popular worldwide [9, 10]. Recently, our research group and others found that orthokeratology controls myopia progression by inhibiting AXL elongation [11–14]. However, the slowing effect of orthokeratology on myopia progression varies from individual to individual [7], and there have been controversies on the mechanism underlying the inhibition of myopia progression in orthokeratology wearers. Although some studies have proposed that peripheral myopic defocus induced by orthokeratology is the main mechanism explaining slowed myopia progression in orthokeratology wearers [15–17], others have found no significant effect of myopic defocus on AXL elongation [18, 19]. Meanwhile, additional factors including changes in higher-order aberrations (HOAs) were also suggested as possible mechanisms [20, 21]. In accordance with this concept, recent reports indicated that the amounts of HOAs are negatively associated with myopia progression during the natural course of axial eye growth in childhood [22, 23]. Considering individual variability in the effects of orthokeratology on myopia progression and controversies regarding the precise underlying mechanism, in the present study, we sought to elucidate the factors affecting AXL growth and myopia progression in orthokeratology.

## Patients and methods

### Study design and patients

The subjects of this prospective study were enrolled from the contact lens clinic at Samsung Medical Center in Seoul, Republic of Korea between March 2016 and March 2017. Thirty-two eyes of 17 patients who matched the inclusion criteria (Table 1) were enrolled. We excluded subjects with spherical equivalent refractive errors (SERs) of less than −5.00 diopters (D), astigmatism of more than 1.50 D, anisometropia of more than 1.50 D, and subjects who had amblyopia (i.e., a difference of two or more Snellen lines between eyes). This study was approved by the institutional review board (IRB) of Samsung Medical Center (IRB no. 2015-07-052) and all work was carried out in accordance with the tenets of the Declaration of Helsinki. Informed consent was secured in writing from all participants and their guardians after an explanation of the nature and possible consequences of the study was provided.

**Table 1:** Inclusion criteria.

1. Aged 7 to 10 years at baseline
2. No history of orthokeratology or contact lens use
3. Spherical equivalent refractive error from −0.50 D to −5.00 D in both eyes
4. Astigmatism ≤ 1.50 D in both eyes
5. Anisometropia ≤ 1.50 D
6. No strabismus demanding treatment
7. Birth weight ≥ 1,500 g
8. No known ocular, systemic, or neurodevelopmental conditions that might affect refractive development
9. No use of medications that might affect refractive development

### Orthokeratology lenses

We used Paragon CRT (paflufocon D, Dk = 100 barrers) reverse-geometry rigid gas permeable lenses. To select trial lenses, sliding table nomograms provided by the manufacturer were used. All subjects were fitted with lenses based on the findings of ophthalmologic examinations including visual acuity, manifest and cycloplegic refractions, fluorescein patterns on slit-lamp examination, and topographic findings. To make the final lens decisions, we performed overrefraction to determine the back vertex power of the required lenses. The subjects were advised to wear them every night for at least eight consecutive hours.

### Measurements

Subjects underwent a complete ophthalmologic examination including uncorrected visual acuity; best-corrected visual acuity; manifested refraction; cycloplegic refraction; autorefraction (WAM-5500; Shigiya Machinery Works Ltd., Hiroshima, Japan) in central, 30° nasal (N30), and 30° temporal (T30) gazes under cycloplegia; slit-lamp examination for the anterior segment; AXL measurement with IOLMaster (Carl Zeiss, Jena, Germany) in central, N30, and T30 gazes; topographic evaluation using scanning slit topography (Orbscan II; Bausch & Lomb, Rochester, NY, USA) and Scheimpflug imaging topography (Pentacam; Oculus, Wetzlar, Germany); wavefront assessment for a 6-mm pupil using a WASCA aberrometer (Carl Zeiss, Jena, Germany) following pupil dilation using a mixture of 0.5% phenylephrine and 0.5% tropicamide (Mydrin-P; Santen Pharmaceutical, Osaka, Japan); and evaluation of the corneal endothelium via noncontact specular microscopy (SP-8000; Konan Medical, Nishinomiya, Japan). Subjects were evaluated one day after the beginning of treatment; one week, one month, and three months after initial lens wear; and every three months afterwards. Autorefraction and AXL measurements in central, nasal, and temporal gazes; corneal topography; and wavefront assessment were repeated at three, six, and 12 months after initial lens wear; orthokeratology lenses were replaced according to visual acuity and refraction.

### Statistical analysis

Data from both eyes were analyzed using a generalized estimation equation (GEE) model, considering the possible correlation between bilateral eyes. Clinical and optical parameters related to AXL growth were evaluated with univariate and multivariate GEE regression analyses: we first performed univariate GEE analysis and then conducted multivariate GEE analysis using only the covariates with *p*-values of less than 0.05 in the univariate modeling stage. *P*-values of less than 0.05 were considered to be statistically significant. Statistical analyses were performed using R 3.4.4 (R Foundation for Statistical Computing, Vienna, Austria).

## Results

This study included 32 eyes of 17 orthokeratology lens–treated myopic children. Table 2 presents the main baseline parameters of the children. The age at initial lens wear was 8.63 years ± 0.83 years. The mean logarithm of the minimum angle of resolution uncorrected visual acuity (UCVA) and best-corrected visual acuity (BCVA) were 0.72 ± 0.32 and 0.01 ± 0.02, respectively. The baseline SERs in the central, N30, and T30 gazes were −2.43 ± 0.97 D, −2.17 ± 0.99 D, and −2.63 ± 0.79 D, respectively. The values of parameters at one year of follow-up and the changes of these parameters between baseline and one year of follow-up are presented in Tables 3 and 4. The spherical equivalent refractive error measured by manifest refraction at one year of follow-up was −0.87 ± 0.64 D.

**Table 2:** Baseline parameters and the relationships between these parameters and AXL growth in a univariable GEE model.

Parameter	Value	Beta	95% CI	*p*-value
Age at initial lens wear (years)	8.63 ± 0.83	-0.054	-0.125–0.017	0.139
Male sex (%)	35.29	0.047	-0.097–0.190	0.525
UCVA (logMAR)	0.72 ± 0.32	0.131	-0.263–0.525	0.515
BCVA (logMAR)	0.01 ± 0.02	1.518	-0.859–3.894	0.211
Axial length (mm)				
Central [C]	24.21 ± 0.60	-0.022	-0.221–0.177	0.831
Nasal 30° [N30]	23.16 ± 0.76	0.068	-0.055–0.191	0.277
Temporal 30° [T30]	22.95 ± 0.66	0.008	-0.101–0.117	0.886
C–N30	1.05 ± 0.49	-0.144	-0.272 –-0.016	0.027*
C–T30	1.25 ± 0.33	-0.037	-0.105–0.031	0.288
Refractive error [SE] (D)				
Manifest refraction	-2.59 ± 0.99	0.064	0.006–0.122	0.030*
Cycloplegic autorefraction				
Central [C]	-2.43 ± 0.97	0.058	-0.013–0.129	0.107
Nasal 30° [N30]	-2.17 ± 0.99	-0.023	-0.100–0.053	0.549
Temporal 30° [T30]	-2.63 ± 0.79	0.035	-0.014–0.084	0.159
N30 –C	0.27 ± 0.59	-0.043	-0.128–0.041	0.317
T30 –C	-0.19 ± 0.46	-0.016	-0.070–0.039	0.571
Topographical values by Orbscan II				
Kmax (D)	44.03 ± 1.39	-0.054	-0.150–0.041	0.262
Kmin (D)	42.88 ± 1.21	-0.022	-0.115–0.071	0.640
Sim K’s astigmatism (D)	1.14 ± 0.45	-0.091	-0.203–0.021	0.110
3-mm-zone irregularity	1.13 ± 0.40	-0.022	-0.069–0.024	0.351
5-mm-zone irregularity	1.46 ± 0.49	-0.017	-0.069–0.035	0.525
Anterior chamber depth (mm)	3.09 ± 0.18	-0.143	-0.363–0.078	0.205
White-to-white (mm)	11.60 ± 0.34	-0.014	-0.075–0.046	0.423
Pupil diameter (mm)	4.54 ± 0.61	0.012	-0.027–0.051	0.541
Central corneal thickness (μm)	541.69 ± 24.34	0.001	-0.001–0.003	0.377
Topographical values by Pentacam				
Anterior Kmax (D)	44.02 ± 1.30	-0.035	-0.092–0.023	0.234
Anterior Kmin (D)	42.72 ± 1.17	-0.014	-0.110–0.081	0.769
Anterior astigmatism (D)	1.30 ± 0.64	-0.020	-0.040–0.000	0.054
Posterior Kmax (D)	-6.52 ± 0.22	0.176	-0.068–0.419	0.157
Posterior Kmin (D)	-6.13 ± 0.19	0.110	-0.169–0.389	0.439
Posterior astigmatism (D)	-0.39 ± 0.15	0.048	-0.046–0.142	0.315
Aberrometric values (RMS, μm)				
Total aberrations	2.63 ± 1.00	-0.040	-0.100–0.020	0.196
Total HOAs	0.17 ± 0.07	0.162	-0.356–0.680	0.539
C_2_^−2^	0.26 ± 0.15	-0.115	-0.300–0.070	0.222
C_2_^0^	4.51 ± 1.81	-0.021	-0.056–0.014	0.244
C_2_^2^	0.72 ± 0.62	-0.042	-0.089–0.005	0.082
C_3_^−3^	0.13 ± 0.12	0.088	-0.122–0.298	0.411
C_3_^−1^	0.13 ± 0.12	-0.050	-0.217–0.117	0.556
C_3_^1^	0.20 ± 0.19	0.028	-0.068–0.123	0.573
C_3_^3^	0.17 ± 0.12	0.043	-0.201–0.287	0.729
C_3_^−1^ + C_3_^1^	0.27 ± 0.18	-0.007	-0.095–0.081	0.882
C_4_^−4^	0.07 ± 0.05	0.092	-0.264–0.448	0.612
C_4_^−2^	0.06 ± 0.04	0.249	-0.555–1.052	0.544
C_4_^0^	0.16 ± 0.13	0.067	-0.159–0.293	0.563
C_4_^2^	0.07 ± 0.07	0.036	-0.385–0.456	0.868
C_4_^4^	0.07 ± 0.05	0.032	-0.408–0.471	0.888
C_2_^−2^ + C_2_^0^ + C_2_^2^	4.63 ± 1.78	-0.024	-0.060–0.012	0.197
C_3_^−3^ + C_3_^1^ + C_3_^1^ + C_3_^3^	0.39 ± 0.17	0.068	-0.128–0.263	0.496
C_4_^−4^ + C_4_^−2^ + C_4_^0^ + C_4_^2^ + C_4_^4^	0.24 ± 0.12	0.093	-0.218–0.404	0.557
Specular microscopic values				
Endothelial cell density (cells/mm^2^)	3216 ± 244	-0.000	-0.000–0.000	0.792
CV of cell area (%)	45.19 ± 7.26	-0.000	-0.005–0.005	0.998
Hexagonality (%)	50.59 ± 10.64	0.002	-0.001–0.006	0.223

All values except for sex are presented in the format of mean ± standard deviation. UCVA, uncorrected visual acuity; logMAR, logarithm of the minimum angle of resolution; BCVA, best-corrected visual acuity; SE, spherical equivalent refractive error; D, diopters; Kmax, maximal keratometric value; Kmin, minimal keratometric value; RMS, root mean square; HOAs, higher-order aberrations; CV, coefficient of variation.

*Statistically significant (*P*-value < 0.05).

**Table 3:** Parameters at one year of follow-up and the relationships between these parameters and AXL growth in a univariable GEE model.

Parameter	Value	Beta	95% CI	*p*-value
Axial length (mm)				
Central [C]	24.60 ± 0.58	0.774	-0.134–1.682	0.095
Nasal 30° [N30]	23.62 ± 0.74	0.054	-0.012–0.121	0.109
Temporal 30° [T30]	23.33 ± 0.67	0.024	-0.038–0.086	0.452
C–N30	0.98 ± 0.48	-0.018	-0.068–0.031	0.467
C–T30	1.26 ± 0.29	0.045	-0.071–0.161	0.445
Refractive error [SE] (D)				
Manifest refraction	-0.87 ± 0.64	-0.008	-0.069–0.053	0.803
Topographical values by Orbscan II				
Kmax (D)	42.54 ± 1.16	-0.066	-0.153–0.021	0.139
Kmin (D)	41.42 ± 1.14	-0.046	-0.130–0.038	0.284
Sim K’s astigmatism (D)	1.13 ± 0.44	-0.053	-0.168–0.062	0.367
3-mm-zone irregularity	2.16 ± 1.01	0.003	-0.026–0.032	0.845
5-mm-zone irregularity	2.96 ± 1.37	0.007	-0.009–0.022	0.407
Anterior chamber depth (mm)	3.10 ± 0.20	0.044	-0.370–0.457	0.836
White-to-white (mm)	11.56 ± 0.38	-0.015	-0.189–0.159	0.867
Pupil diameter (mm)	4.75 ± 0.69	0.046	-0.009–0.102	0.103
Central corneal thickness (μm)	543.59 ± 27.04	0.002	-0.001–0.004	0.273
Topographical values by Pentacam				
Anterior Kmax (D)	42.48 ± 0.95	-0.005	-0.066–0.056	0.878
Anterior Kmin (D)	41.23 ± 0.98	0.005	-0.044–0.053	0.855
Anterior astigmatism (D)	1.25 ± 0.44	-0.015	-0.055–0.025	0.459
Posterior Kmax (D)	-6.48 ± 0.19	-0.009	-0.271–0.254	0.949
Posterior Kmin (D)	-6.13 ± 0.15	-0.112	-0.371–0.148	0.399
Posterior astigmatism (D)	-0.36 ± 0.12	0.060	-0.089–0.210	0.430
Aberrometric values (RMS, μm)				
Total aberrations	1.85 ± 0.82	0.050	-0.011–0.111	0.111
HOAs	0.34 ± 0.12	-0.266	-0.611–0.080	0.131
C_2_^−2^	0.56 ± 0.39	-0.070	-0.146–0.006	0.072
C_2_^0^	2.99 ± 1.51	0.028	-0.006–0.062	0.104
C_2_^2^	0.97 ± 0.60	0.028	-0.043–0.100	0.437
C_3_^−3^	0.18 ± 0.15	0.084	-0.217–0.386	0.582
C_3_^−1^	0.44 ± 0.35	-0.123	-0.272–0.025	0.104
C_3_^1^	0.31 ± 0.27	0.001	-0.102–0.104	0.985
C_3_^3^	0.19 ± 0.12	-0.122	-0.325–0.081	0.239
C_3_^−1^ + C_3_^1^	0.59 ± 0.36	-0.107	-0.210 –-0.005	0.040*
C_4_^−4^	0.08 ± 0.05	0.061	-0.644–0.766	0.865
C_4_^−2^	0.11 ± 0.10	0.003	-0.275–0.282	0.980
C_4_^0^	0.48 ± 0.24	-0.036	-0.159–0.087	0.568
C_4_^2^	0.15 ± 0.11	0.071	-0.293–0.436	0.701
C_4_^4^	0.10 ± 0.08	-0.319	-0.742–0.103	0.138
C_2_^−2^ + C_2_^0^ + C_2_^2^	3.31 ± 1.43	0.030	-0.008–0.067	0.123
C_3_^−3^ + C_3_^1^ + C_3_^1^ + C_3_^3^	0.67 ± 0.36	-0.108	-0.210 –-0.006	0.039*
C_4_^−4^ + C_4_^-2^ + C_4_^0^ + C_4_^2^ + C_4_^4^	0.56 ± 0.22	-0.046	-0.184–0.092	0.515
Specular microscopic values				
Endothelial cell density (cells/mm^2^)	3194 ± 279	-0.000	-0.000–0.000	0.061
CV of cell area (%)	46.84 ± 8.03	0.002	-0.003–0.008	0.389
Hexagonality (%)	48.09 ± 8.67	-0.002	-0.006–0.002	0.310

All values are presented in the format of mean ± standard deviation. UCVA, uncorrected visual acuity; logMAR, logarithm of the minimum angle of resolution; BCVA, best-corrected visual acuity; SE, spherical equivalent refractive error; D, diopters; Kmax, maximal keratometric value; Kmin, minimal keratometric value; RMS, root mean square; HOAs, higher-order aberrations; CV, coefficient of variation.

*Statistically significant (*P*-value < 0.05).

**Table 4:** Changes in parameters between baseline and one year of follow-up and the relationships between these changes and AXL growth in a univariable GEE model.

Parameter	Value	Beta	95% CI	*p*-value
Axial length (mm)				
Δ Central [C]	0.28 ± 0.18			NA
Δ Nasal 30° [N30]	0.34 ± 0.40	0.023	-0.018–0.064	0.273
Δ Temporal 30° [T30]	0.29 ± 0.34	0.017	-0.006–0.039	0.140
Δ C–N30	0.06 ± 0.38	-0.033	-0.118–0.052	0.443
Δ C–T30	-0.25 ± 0.43	-0.080	-0.218–0.059	0.259
Topographical values by Orbscan II				
Δ Kmax (D)	-1.49 ± 0.78	-0.019	-0.075–0.037	0.514
Δ Kmin (D)	-1.47 ± 0.78	-0.022	-0.058–0.014	0.227
Δ Sim K’s astigmatism (D)	0.00 ± 0.43	0.008	-0.049–0.064	0.793
Δ 3 mm zone irregularity	1.03 ± 0.98	0.012	-0.015–0.038	0.384
Δ 5 mm zone irregularity	1.50 ± 1.31	0.009	-0.002–0.021	0.121
Δ Anterior chamber depth (mm)	0.01 ± 0.07	0.289	-0.148–0.726	0.195
Δ White-to-white (mm)	-0.04 ± 0.08	0.006	-0.012–0.024	0.376
Δ Pupil diameter (mm)	0.21 ± 0.60	0.046	-0.009–0.102	0.103
Δ Central corneal thickness (μm)	1.91 ± 9.70	0.001	-0.002–0.003	0.680
Topographical values by Pentacam				
Δ Anterior Kmax (D)	-1.38 ± 0.68	0.017	-0.001–0.036	0.060
Δ Anterior Kmin (D)	-1.35 ± 0.79	-0.015	-0.037–0.007	0.189
Δ Anterior astigmatism (D)	-0.04 ± 0.77	0.012	0.004–0.019	0.002*
Δ Posterior Kmax (D)	0.01 ± 0.09	-0.095	-0.275–0.084	0.297
Δ Posterior Kmin (D)	-0.01 ± 0.10	-0.081	-0.297–0.135	0.462
Δ Posterior astigmatism (D)	0.02 ± 0.16	0.010	-0.141–0.160	0.898
Aberrometric values (RMS, μm)				
Δ Total aberrations	-0.82 ± 1.14	0.044	-0.003–0.085	0.060
Δ HOAs	0.18 ± 0.14	-0.257	-0.584–0.070	0.124
Δ C_2_^−2^	0.31 ± 0.37	-0.066	-0.133–0.001	0.054
Δ C_2_^0^	-1.57 ± 2.13	0.029	-0.000–0.057	0.050
Δ C_2_^2^	1.63 ± 1.21	0.005	-0.048–0.058	0.855
Δ C_3_^−3^	0.04 ± 0.16	-0.004	-0.132–0.124	0.950
Δ C_3_^−1^	0.32 ± 0.37	-0.103	-0.219–0.014	0.084
Δ C_3_^1^	0.10 ± 0.33	-0.024	-0.110–0.061	0.580
Δ C_3_^3^	0.01 ± 0.13	-0.062	-0.235–0.112	0.485
Δ (C_3_^−1^ + C_3_^1^)	0.33 ± 0.42	-0.105	-0.201 –-0.009	0.032*
Δ C_4_^−4^	0.01 ± 0.07	-0.033	-0.269–0.203	0.784
Δ C_4_^−2^	0.06 ± 0.10	-0.089	-0.366–0.187	0.525
Δ C_4_^0^	0.34 ± 0.25	-0.032	-0.147–0.083	0.582
Δ C_4_^2^	0.09 ± 0.12	0.034	-0.262–0.330	0.822
Δ C_4_^4^	0.03 ± 0.09	-0.224	-0.533–0.086	0.157
Δ (C_2_^−2^ + C_2_^0^ + C_2_^2^)	-1.38 ± 1.98	0.032	0.002–0.062	0.038*
Δ (C_3_^−3^ + C_3_^1^ + C_3_^1^ + C_3_^3^)	0.29 ± 0.40	-0.114	-0.221 –-0.008	0.036*
Δ (C_4_^−4^ + C_4_^-2^ + C_4_^0^ + C_4_^2^ + C_4_^4^)	0.35 ± 0.25	-0.044	-0.171–0.082	0.489

All values are presented in the format of mean ± standard deviation. UCVA, uncorrected. visual acuity; logMAR, logarithm of the minimum angle of resolution; BCVA, best-corrected visual acuity; SE, spherical equivalent refractive error; D, diopters; Kmax, maximal keratometric value; Kmin, minimal keratometric value; RMS, root mean square; HOAs, higher-order aberrations.

*Statistically significant (*P*-value < 0.05).

The baseline AXLs in the central, N30, and T30 gazes were 24.32 ± 0.60 mm, 23.28 ± 0.77 mm, and 23.05 ± 0.64 mm, respectively, while the AXLs at one year of follow-up were 24.60 ± 0.58 mm, 23.62 ± 0.74 mm, and 23.33 ± 0.67 mm, respectively. The amount of changes in the three gazes during one year were 0.28 ± 0.18, 0.34 ± 0.40, and 0.29 ± 0.34, respectively.

In Tables 2, 3 and 4, univariable GEE analyses demonstrate the influence of the parameters of baseline and at one year follow-up as well as the changes between baseline and one year of follow-up with respect to AXL growth. In the analyses, the baseline difference in AXL between C and N30; baseline manifest SER; posttreatment difference in SER between C and N30; posttreatment coma (C_3_^−1^ + C_3_^1^) and third-order HOAs (C_3_^−3^ + C_3_^1^ + C_3_^1^ + C_3_^3^); the changes between baseline and one year of follow-up for coma, second-order aberrations (C_2_^−2^ + C_2_^0^ + C_2_^2^), and third-order HOAs; and the change in anterior corneal astigmatism were significantly associated with AXL change.

**Fig 1:**
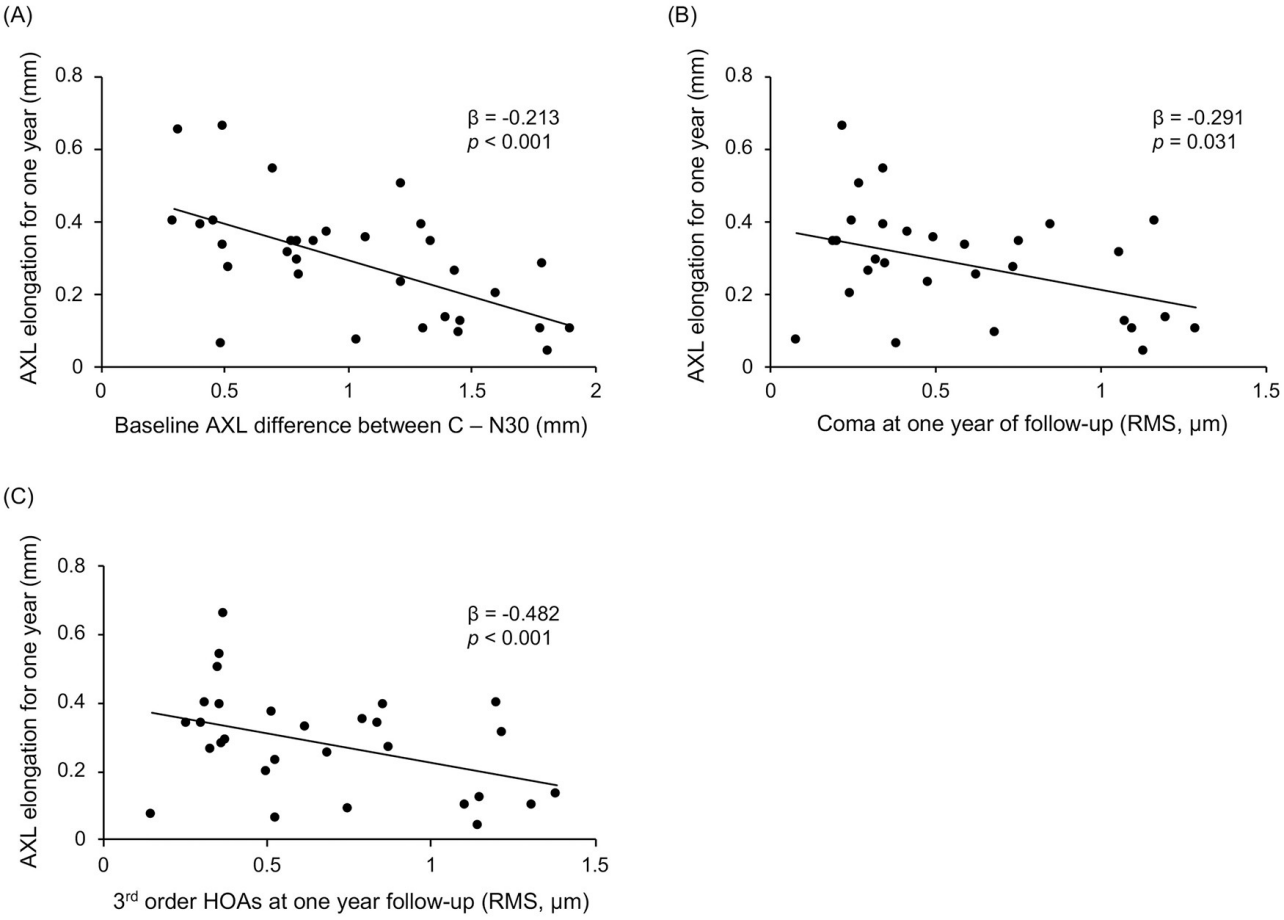
Scatterplots showing the relationships between (A) baseline difference in AXL between C and N30, (B) posttreatment coma, and (C) third-order HOAs with AXL elongation for one year. All of these parameters were negatively correlated with AXL elongation. AXL, axial length; C, central gaze; N30, nasal 30° gaze; RMS, root mean square; HOAs, higher-order aberrations.

When all parameters revealed by univariable GEE analyses to be significantly associated with AXL growth were entered into the multivariable-adjusted GEE analyses and simultaneously adjusted, the baseline difference in AXL between C and N30 (β = −0.213, *p* < 0.001); baseline manifest SER (β = −0.040, *p* = 0.033); posttreatment coma (β = −0.291, *p* = 0.031) and third-order HOAs (β = −0.482, *p* < 0.001); and change in second-order aberrations at one year of follow-up (β = 0.025, *p* = 0.027) were identified as significant factors (Table 5). Fig 1 shows the relationships between AXL growth and these significant factors.

**Table 5:** The relationships between parameters with statistically significant associations on univariable analysis and AXL growth in a multivariable GEE model.

Parameter	Value	Beta	95% CI	*p*-value
Baseline axial length (Central–Nasal 30°, mm)				
Univariable analysis	1.05 ± 0.49	-0.144	-0.272 –-0.016	0.027*
Multivariable analysis		-0.213	-0.257 –-0.168	0.000*
Baseline manifest refraction [SE] (D)				
Univariable analysis	-2.59 ± 0.99	0.064	0.006–0.122	0.030*
Multivariable analysis		-0.040	-0.078 –-0.003	0.033*
Refractive error (Nasal 30°–Central) at one year of follow-up [SE] (D)				
Univariable analysis	-0.53 ± 1.41	0.006	0.001–0.012	0.027*
Multivariable analysis		0.009	-0.015–0.033	0.462
C_3_^−1^ + C_3_^1^ at 1 year follow-up (RMS, μm)				
Univariable analysis	0.59 ± 0.36	-0.107	-0.210 –-0.005	0.040*
Multivariable analysis		-0.291	-0.554 –-0.027	0.031*
C_3_^−3^ + C_3_^1^ + C_3_^1^ + C_3_^3^ at one year of follow-up (RMS, μm)				
Univariable analysis	0.67 ± 0.36	-0.108	-0.210 –-0.006	0.039*
Multivariable analysis		-0.482	-0.573 –-0.390	0.000*
Δ (C_3_^−1^ + C_3_^1^) (RMS, μm)				
Univariable analysis	0.33 ± 0.42	-0.105	-0.201 –-0.009	0.032*
Multivariable analysis		-0.056	-0.135–0.022	0.158
Δ (C_2_^−2^ + C_2_^0^ + C_2_^2^) (RMS, μm)				
Univariable analysis	-1.38 ± 1.98	0.032	0.002–0.062	0.038*
Multivariable analysis		0.025	0.003–0.047	0.027*
Δ (C_3_^−3^ + C_3_^1^ + C_3_^1^ + C_3_^3^) (RMS, μm)				
Univariable analysis	0.67 ± 0.36	-0.114	-0.221 –-0.008	0.036*
Multivariable analysis		0.183	-0.116–0.298	0.320
Δ Anterior astigmatism by Pentacam (D)				
Univariable analysis	-0.04 ± 0.77	0.012	0.004–0.019	0.002*
Multivariable analysis		0.019	-0.029–0.068	0.397

All values are presented in the format of mean ± standard deviation. UCVA, uncorrected. visual acuity; logMAR, logarithm of the minimum angle of resolution; BCVA, best-corrected visual acuity; SE, spherical equivalent refractive error; D, diopters; Kmax, maximal keratometric value; Kmin, minimal keratometric value; RMS, root mean square; CV, coefficient of variation.

*Statistically significant (*P*-value < 0.05).

## Discussion

In this study, we demonstrated that the baseline differences in AXL between C and N30 and posttreatment ocular HOAs are significantly associated with AXL growth and myopia progression in orthokeratology. Considering the interindividual variation in myopia progression after orthokeratology wear, our results would be beneficial in the selection of suitable candidates for orthokeratology to inhibit myopic progression as well as correct myopia.

In the past few decades, the use of orthokeratology treatment worldwide has become more popular because of its effectiveness in the correction of myopia and the resolution of associated safety issues [9]. Ever since orthokeratology has been reported to inhibit myopia progression [11–14], studies have suggested possible underlying mechanisms of the slowed myopia progression yielded by orthokeratology. Among them, peripheral myopization induced by anterior corneal reshaping from prolate to spherical or even oblate after orthokeratology treatment has been suggested as a plausible hypothesis [7, 24]. Regarding potential hypotheses, previous studies [16, 17] mostly focused on the analysis of changes in peripheral refraction after orthokeratology treatment. Following the relative myopic change of peripheral refraction as compared with central refraction after orthokeratology lens wear demonstrated by Queiros et al. [16], Kang et al. separately reported that orthokeratology in one eye induced relative peripheral myopia, while rigid gas-permeable lens wear in the contralateral eye prompted no change in peripheral refraction [17]. However, these authors did not provide any direct evidence regarding whether the extent of peripheral myopization is significantly correlated with AXL growth rate or not. In the present study, we demonstrated that a subject with a relatively large baseline difference between central and nasal AXLs was more susceptible to orthokeratology treatment considering slower myopia progression after the treatment versus those with smaller baseline differences. We postulate that this is because a myopic eye with more hyperopic peripheral defocus prior to orthokeratology lens wear has a higher potential to be shifted to relative myopic peripheral defocus. Previously, to retrospectively evaluate the effects of peripheral refraction on AXL elongation in orthokeratology-treated eyes, our group used topographically measured values of peripheral refraction because we did not have directly measured peripheral AXL data [25]. In this study, to the best of our knowledge, we have provided the first evidence that higher baseline differences between central and peripheral AXLs are significantly associated with lower AXL elongation.

Increased HOAs has been proposed as a possible underlying mechanism of inhibited myopia progression in orthokeratology lens wear. By using multivariate analysis, Hiraoka et al. [20, 21] demonstrated that the larger the change in coma-like HOAs (third-order Zernike coefficients) or the smaller the change in defocus (C_2_^0^), the slower the progression of myopia. In their study, they measured ocular HOAs for a 4-mm pupil without the use of dilating drugs by using a Hartmann–Shack aberrometer. Meanwhile, the relationship between the posttreatment values of HOAs and myopia progression was not evaluated by them. Similarly, in the present study, multivariate GEE analysis demonstrated that coma (C_3_^−1^ + C_3_^1^) and third-order HOAs (C_3_^−-3^ + C_3_^1^ + C_3_^1^ + C_3_^3^) at one year of follow-up and changes in second-order aberrations (C_2_^−2^ + C_2_^0^ + C_2_^2^) between baseline and one year of follow-up were significantly associated with AXL elongation and myopia progression. In particular, third-order HOAs (β = −0.482) and coma (β = −0.291) at one year of follow-up were more relevant factors affecting AXL elongation versus changes in second-order aberrations between baseline and one year of follow-up (β = 0.032). This result implies that asymmetric optical changes by asymmetric third-order HOAs such as coma and trefoil could be associated with AXL elongation. We assume that the identification of this similar but different result as compared with that of previous report [21] could be attributed to the fact that we performed the aberrometry measurements after pharmacologically induced mydriasis because the changes in ocular HOAs are very dynamic and largely influenced by various factors such as pupil size and accommodation status [26, 27]. Meanwhile, apart from analyses of the relationship between HOAs and myopia progression in orthokeratology lens wear, recent landmark studies conducted on myopic children wearing single-vision spectacles have also shown a significant association between the amount of HOAs and axial elongation [22, 23]. Cho and colleagues showed that total ocular HOAs, spherical aberrations (C_4_^0^ and C_4_^0^ + C_6_^0^) and trefoils (C_3_^−-3^ and C_3_^3^) were negatively associated with axial elongation [23]. In addition, Hiraoka and colleagues showed negative association between total corneal HOAs and axial elongation after adjusting potential confounding factors [22]. Overall, these findings suggested that HOAs has a substantial role in myopia progression in children wearing orthokeratology lenses or single-vision spectacles.

The major strength of this study was that we determined the exact relationship between peripheral myopization and myopia progression in orthokeratology lens wear by evaluating the relationship of baseline difference between central and nasal AXL with axial elongation. Thus, our study demonstrates a substantive degree of significance by proving the peripheral myopization hypothesis and providing eligible criteria for the selection of subjects for orthokeratology treatment in the context of the inhibition of myopia progression. Also, we verified a previously reported influence of ocular HOAs on AXL elongation in orthokeratology lens wear by performing aberrometry measurements after pupil dilation and wavefront assessments for a 6-mm pupil, solidifying the possibility of ocular HOAs as a putative mechanism of slower myopia progression in orthokeratology lens wear. However, the present study also had limitations, including a short-term follow-up and a small sample size.

In conclusion, we confirm and solidify certain underlying mechanistic bases including peripheral myopization and asymmetric optical changes in orthokeratology treatment for the inhibition of myopia progression, suggesting appropriate criteria to assist with candidate selection to inhibit myopia progression.

## Supporting information

S1 Dataset Data analyzed. (XLSX)
